# Headache yesterday in Russia: its prevalence and impact, and their application in estimating the national burden attributable to headache disorders

**DOI:** 10.1186/1129-2377-16-7

**Published:** 2015-01-20

**Authors:** Ilya Ayzenberg, Zaza Katsarava, Asya Sborowski, Mark Obermann, Michail Chernysh, Vera Osipova, Guzelya Tabeeva, Timothy J Steiner

**Affiliations:** Department of Neurology, University of Bochum, Bochum, Germany; Department of Neurology, University of Essen, Essen, Germany; Institute of Sociology, Russian Academy of Sciences, Moscow, Russia; Department of Neurology, First Moscow State Medical University, Moscow, Russia; Moscow Research Clinical Center for Neuropsychiatry, Moscow, Russia; Department of Neuroscience, Norwegian University of Science and Technology, Trondheim, Norway; Division of Brain Sciences, Imperial College London, London, UK

**Keywords:** Headache yesterday, Prevalence, Socioeconomic burden, Lost productivity, Cost of illness, Global Campaign against headache

## Abstract

**Background:**

Evaluation of the prevalence and impact of headache on the preceding day (“headache yesterday”; HY) is a new approach, allowing more precise estimation of headache-attributed burden without recall error. The aim of the study was to estimate the national burden attributable to headache disorders in Russia by applying measures of prevalence of HY and its impact on productivity and daily activities in the general population.

**Methods:**

We interviewed a representative population-based sample face-to-face by visiting randomly selected households throughout Russia. We randomly selected one adult aged 18–65 years from each. We followed a structured questionnaire including diagnostic questions, enquiry into occurrence of HY and various aspects of attributed burden.

**Results:**

Participation rate was 74.3%. One in seven participants (14.5%; men 9.1%: women 19.3%) reported HY. Approximately half of these had one of the subtypes of headache occurring on ≥15 days/month; the remainder had episodic migraine or tension-type headache almost equally. Mean duration of headache was 6.0 ± 4.4 hours. In 88.3% headache intensity was moderate or severe (mean 2.1 on a scale 1–3) and in 73.9% HY impaired daily activity. Loss of productivity at work due to headache totalled 2.6 million person-years/year, or 4.0% of workforce capacity. This estimate exceeded by 70% a previous estimate from the same survey based on recall over the preceding 3 months. There was greater impact on other daily activities.

**Conclusion:**

Recall-error-free estimation shows lost productivity every day due to headache in the Russian population is enormously high. Measures to redress these losses – effective structured health-care services supported by educational programmes – should be seen as a public-health priority while almost certainly being cost-saving.

## Background

The Global Burden of Disease Survey 2010 (GBD2010) ranked tension-type headache (TTH) and migraine as the second and third most common diseases worldwide [1]. More importantly, these primary headache disorders are associated with disability, reduced quality of life, public ill health and high economic burdens on both individual and population levels. Migraine has been recognized as the seventh highest among specific causes of disability worldwide [1, 2]. The costs of headache disorders are enormous: estimated in 2012 at well in excess of EUR 100 billion per year in the European Union [3].

In the last 20 years a large number of population-based studies of headache prevalence have been performed, especially in the United States of America and Western Europe [4]. Some have also estimated headache-attributed burden, and those assessing socioeconomic impact have employed questionnaires such as the Migraine Disability Assessment (MIDAS) instrument [5] or its derivative, the Headache-Attributed Lost Time (HALT) questionnaire [5, 6]. The former, and in its original form (HALT-90) also the latter, depend upon enquiry relating to the three months prior to the interview, an approach subject to the limitations and errors (and possible biases) of recall over such a long period [7]. The consequences of these limitations, errors and possible biases need to be considered, and an alternative approach that allows this is enquiry into headache occurring on the day before the interview (“headache yesterday”; HY). Recall of HY and its impact is likely to be highly reliable. While what happened yesterday is not indicative of headache-attributed burden in individuals, at population level it is. Such an approach does, however, require a high number of respondents, since far fewer people have headache yesterday than the number who have headache in 3 months. Furthermore, diagnosis cannot reliably be based on a single headache episode; unless a respondent can report that HY is typical of a recurrent headache that has been diagnosed, its nature will not be known.

A question subset concerning HY [8, 9], has been included into several recent large epidemiological studies supported by *Lifting The Burden* and conducted within the Global Campaign against Headache, including those in China [10], India [11], Pakistan [12], Nepal [13], Zambia (unpublished) and countries of the Eurolight project [7].

Here we report the analysis of HY in Russia. We apply this analysis in making an estimate of the national socioeconomic burden attributable to headache disorders, and compare this to the estimate based on 3-months’ recall [14].

## Methods

The methods of the study have been described in detail previously [15] and are only summarised here.

### Ethics

The study protocol was approved by the ethics committee of the Russian Academy of Sciences. All participants were informed of the purpose of the survey and gave verbal consent.

### Sampling

This nationwide cross-sectional survey of the working-age population (18–65 years) included 44 settlements of six of the seven Federal Districts of Russia. The inhabitants of the seventh, Far-East region (approximately 6% of the total population) were not included owing to its very low population density and geographical seclusion. In order to achieve a representative sample of the country’s population, we performed hierarchical cluster sampling. Within clusters we made a random selection of households, which we visited unannounced, selecting for interview only one person per household, also randomly.

### Enquiry

Face-to-face interviews were conducted by trained non-medical interviewers employed by the Russian Institute of Sociology using a structured questionnaire. To reduce possible interest bias, potential respondents were asked if they would take part in a short interview of the Russian Institute of Sociology and were not informed of the focus of the survey before they agreed (although they could stop the interview later).

The questionnaire developed by *Lifting The Burden* [9] was adapted, translated into the Russian language and validated as reported previously [16]. This modular questionnaire included demographic enquiry and headache screening questions and, for those with headache in the last year, diagnostic questions based on ICHD-II criteria [17] and enquiry into various aspects of headache-attributed burden (including the HALT questionnaire). Questions on HY included duration of HY (in hours up to 24), intensity of HY (graded as “mild”, “moderate” or “severe”), usage of acute medications, lost productivity because of HY (could do “everything”, “more than half”, “less than half” or “nothing” of what had been planned), impact of HY in limiting daily activity (“complete”, “partial” or “none”).

### Statistics

We used statistics descriptively, calculating means, standard deviations (SDs) and medians as appropriate. We used Pearson’s chi-squared to test differences between proportions for significance.

## Results

### Demographic characteristics and 1-year prevalence

Of 2,725 contacted eligible persons, 2,025 (74.3%) agreed to participate in the study and gave consistent answers. Another 588 (21.6%) refused the interview, without getting to know the topic of the survey, and 112 (4.1%) interrupted the interview or gave unusable answers. These were classed as non-participants. The participating sample were aged 39.5 (SD ± 13.4) years, compared with the national population mean of 39.2 years, and 47.4% were male (nationally 46.6%). Other demographic characteristics also revealed no differences between the study sample and the general population [15]. As previously reported, the overall 1-year prevalence of headache was 62.9%: 20.3% fulfilled ICHD-II criteria [17] for episodic migraine (definite or probable), 30.9% for episodic TTH (definite or probable), 10.5% had headache on ≥15 days/month and 1.1% had unclassified episodic headaches [15].

### Headache yesterday

HY was reported by 293 participants, representing a 1-day prevalence of 14.5% (CI 95%: 13.0 - 16.0), and by approximately twice as many women as men (19.3% *vs.* 9.1%). This matched almost exactly the predicted 1-day prevalence of 14.6% based on 1-year prevalence and mean frequency in headache days/month (extrapolated from the last 3 months) (Table 1). HY was reported to be typical of their usual most bothersome (diagnosed) headache by 93.5% of participants (males 95.4%, females 92.7%). Ignoring the diagnoses of the remaining 6.5%, we found HY to be due more or less equally to episodic migraine (24.9%) and episodic TTH (24.2%), each responsible for a quarter of cases and eclipsed by the various causes of headache on ≥15 days/month, which were responsible for twice as many (49.5%; Table 1). Thus migraine and TTH yesterday were each reported by about 3.5% of the total sample, and these numbers also were matched by the predicted 1-day prevalences of these headache types (Table 1).

**Table 1:** **Headache yesterday according to diagnosis, and comparison with predicted 1-day prevalence based on reported frequency and 1-year prevalence**

Headache type	Headache yesterday (HY)		Predicted 1-day prevalence		
	Participants with HY (%)	Total sample (%)	1-year prevalence in sample (%)	Mean headache days/month	Calculated 1-day prevalence (%)
All headache	100.0	14.5	62.9	7.1 ± 8.3	14.6
Episodic migraine	24.9	3.6	20.3	4.4 ± 3.5	2.9
Episodic TTH	24.2	3.5	30.9	3.5 ± 3.1	3.5
Headache on ≥15 days/month	49.5	7.2	10.5	23.1 ± 6.7	8.0

Mean duration of HY was 6.0 ± 4.4 hours. Over half of participants (52.2%) reported headache duration of <4 hours, probably explained by the widespread usage of acute medications: 51.7% of participants with HY (or 7.3% of the total survey population) took such medications (Table 2). Approximately half of these took simple analgesics and another half took combination analgesics; only one of 286 participants reported taking a triptan. Nonetheless, almost one third of participants reported headache effectively lasting throughout the whole day. Headache intensity was moderate or severe in 88.2% of participants; we graded “mild”, “moderate” and “severe” on a scale 1–3, treated these values as continuous data and derived a mean intensity of 2.1 ± 0.6. HY limited daily activities in 73.9% of participants, completely so in 22.9%. Lost productivity due to HY was reported by 69.9% of participants with HY: 24.2% could do less than half of what they had planned and 15.6% could do nothing. The analysis of HALT (and MIDAS) offsets those who could do less than half against those who could do more than half, counting the former as doing nothing and the latter as doing everything [6]. Applying this concept, we found that in total HY cost those affected 39.8% of their productivity yesterday.

**Table 2:** **Burden of headache yesterday by gender**

Burden variable	All	Males	Females
Duration of HY (hr) (mean ± SD; median)	6.0 ± 4.0; 4.0	6.1 ± 4.4; 4.0	5.9 ± 4.4; 4.0
<1 hr (%)	13.0	11.5	13.6
1-4 hr (%)	39.2	42.5	37.9
5-12 hr (%)	16.4	12.6	18.0
>12 hr (%)	31.4	33.3	30.6
**Intensity of HY** **(%)**			
Mild pain	11.7	12.6	11.3
Moderate pain	63.4	61.0	64.5
Severe pain	24.8	26.4	24.1
**Limitation of daily activities (%)**			
None	26.0	28.7	24.9
Partial	51.0	48.3	52.2
Complete	22.9	23.0	22.9
**Lost productivity (%)**			
None	30.1	33.3	28.7
Did >50% of expected	30.1	23.0	33.2
Did <50% of expected	24.2	26.4	23.3
Did nothing	15.6	17.2	14.9
**Used acute medications yesterday (%)**	51.7	50.6	52.2
Simple analgesics	51.3	55.8	49.5
Combination analgesics	48.0	44.1	49.5
caffeine-containing	48.0	44.1	49.5
codeine-containing	16.9	16.8	17.1
barbiturate-containing	15.5	16.8	15.2
Triptans	0.7	0	1.0

Despite the two-fold difference in prevalence, we saw no significant differences in headache duration or intensity, in functional impairment or lost productivity, or in use of medication between men and women (Table 2).

Although all 2,025 participants were of working age, some were students (n = 100), some retired (n = 237) and some gave no answer concerning their employment status (n = 85). Lost productivity was especially high (54.0%) among retired participants with HY. For purposes of analysis we regarded all 1,603 other participants as potential workers, and categorized them as employed for money (including self-employed; n = 1,356) or unemployed (including housewives; n = 247) (Table 3). We analyzed lost productivity separately for employed and unemployed participants, and, in the case of the former, for those for whom yesterday had been a workday (54.9%) or a free day (45.1%). There were differences, though not statistically significant: 11.9% of employed participants reported HY (11.6% with a workday, 12.4% with a free day yesterday) compared with 15.4% of unemployed (Pearson’s chi-squared = 2.26; P = 0.13). Among those with HY for whom yesterday had been a workday, 34.1% of productivity was lost (23.9% able to perform less than half and 10.2% nothing because of HY). Approximately one third (30.7%) could do everything and another third (35.2%) more than half of what they had planned. Total per-person lost productivity was comparable in those employed who had a workday (34.1%) or free day (35.2%) yesterday and those unemployed (36.9%); however, those with a free day were slightly, and those unemployed very markedly, more likely to report that they did nothing rather than less than half (Table 3).

**Table 3:** **Lost productivity attributed to headache yesterday (HY) by employment**

	All potential workers*	All employed participants	Employed with workday yesterday	Employed with free day yesterday	Unemployed
	(N = 1,603)	(n = 1,356)	(n = 768)	(n = 588)	(n = 247)
**Reporting HY,** n (%)	200 (12.5)	162 (11.9)	89 (11.6)	73 (12.4)	38 (15.4)
**Lost productivity due to HY (%)**					
none (did everything as planned)	34.5	34.0	30.7	38.0	36.8
did >50% of expected	30.5	31.4	35.2	26.8	26.3
did <50% of expected	20.8	23.3	23.9	22.5	10.5
did nothing	14.2	11.3	10.2	12.7	26.3

*The term “potential workers” excludes students, retirees and participants who gave no answer concerning their employment status (see text).

We calculated headache-attributed lost productivity among those who worked yesterday as the product of per-person loss productivity (34.1%) and prevalence of HY (11.6%), arriving at 4.0%, representing the lost productivity in the workforce on *every* workday. Using the same approach for participants for whom yesterday was not a workday (employed with a free day, or unemployed), we discovered higher losses: 4.4% and 5.7% respectively. In other words, less productivity was lost from worktime than from household work and other activities.

The two-fold difference in prevalence between genders remained stable in employed participants with a workday yesterday (women 16.4%, men 7.5%).

## Discussion

This is the third published analysis of headache yesterday (HY) worldwide, following those in China [10] and eight countries of Europe [7], and the first in Russia. It is of some interest to know the proportion of a population afflicted by headache on any day, but the true value of this population-based enquiry lies in its almost complete detachment from the recall errors to which traditional burden-of-headache surveys are so strongly subject. This approach was pioneered by *Lifting The Burden* in its Global Campaign against Headache [18, 19] and is included in the Headache-Attributed Restriction, Disability, Social Handicap and Impaired Participation (HARDSHIP) questionnaire recently developed for population-based studies of headache [8, 9].

In our study, 14.5% of participants, aged 18–65 years (*i.e.*, of working age), reported HY. Extrapolated to the entire population, this means that approximately every seventh person of working age in the country suffers from headache on any and every day. This finding is quite similar to the 15-19% reported from 15 countries in Europe [7], although these data from the Eurolight study were not entirely population-based. In absolute numbers, in Russia’s total population of 142 million, there are 99.8 million adults aged 18–65 years [20] among whom, on any and every day, there are 14.5 million with headache.

Comparing these data with those previously reported in our 1-year headache prevalence study [15], we found by prediction the same result – 14.6% of the adult population with headache every day. Interestingly, in both earlier published studies of HY, similar internal consistency was seen between reported HY and predicted 1-day prevalence of headache based on recalled frequency during the preceding 3 months [7, 10]. In our study, the fact that well over 90% of participants reported HY to be typical of their most bothersome (and therefore diagnosed) headache allowed us to analyze HY according to headache type. For each – episodic migraine, episodic TTH and headache on ≥15 days/month – quite similar consistency was observed.

Headache on ≥15 days/month is highly prevalent (10.5%) in Russia [15]. In accordance with this, the various causes of headache on ≥15 days/month accounted for almost 50% of cases of HY, confirming their importance as contributors to population ill health and disability in Russia [14]. Almost 52% of those with HY took acute headache medications, which extrapolates to 7.5 (14.5 × 0.52) million people doing so every day. Almost half of these (48%, extrapolating to 3.6 million people) used combination analgesics, medications probably closely associated with medication-overuse headache (MOH) [21]. In Russia, medication overuse was also reported by two thirds of survey participants with headache on ≥15 days/month [15], and their diagnosis was probable MOH.

The main purpose of this survey was a correct evaluation of headache-attributed burden. The mean duration of HY of 6 hours was relatively short – reflecting probably the effect of treatment – but in line with duration reported in the European study [7] and slightly more than was found in China [10]. From this mean duration and the prevalence of HY, we calculate that 3.6% of the population aged 18–65, or 3.6 million adults, have headache at any one time. This proportion is quite similar to the 4.0% in Europe (6) but double the 1.8% reported in China [10]. In terms of pain intensity, the mean was 2.1 on a scale of 1–3, expected to be disabling. A quarter of participants (24.8%) described severe headache, and approximately the same proportion (22.9%) reported that their daily activities were reduced to nil by HY (Table 2). In Russia’s 99.8 million people of working age [20], this means about 3.5 million people so affected every day. Since the indirect costs make up by far the greater part of all headache-attributed costs [3, 22], and in order accurately to evaluate headache-attributed economic burden, we concentrated on the working population. In employed participants, the prevalence of HY was lower (11.9%) without meaningful difference between those who had a workday (11.6%) or a free day (12.4%) yesterday, indicating that the phenomenon of “weekend headache” has a minor effect at population level. Above, we calculated a 4.0% lost productivity in the workforce on every workday because of headache (see Results). Since the total working population aged 18–65 years in Russia is 65.6 million [20], this means 2.62 million people unproductive every day or a loss of 2.62 million person-years/year. This number is approximately 70% higher than the 1.54 million person-years/year estimated through recall over the last 3 months [14]. Even more time was lost to HY from household work or leisure. Household work is generally more easily abandoned or delayed than paid labour, so this finding was expected, and has been reported in previous studies [14, 23].

Although in Table 1 we present HY according to diagnosis (based on participants’ assertions in 93.5% of cases that HY was typical of their diagnosed most bothersome headache), because of the diagnostic uncertainty we did not go beyond this to attribute burden to each headache type. In terms of prevalence, approximately half of cases of HY were one or another of the causes of headache on ≥15 days/month and a quarter were each of migraine and TTH. *Burden* would depend, in particular, on how disabling each headache type tended to be, with, on average, migraine expected to be most and TTH least. Headache on ≥15 days/month is variable in its effect; although chronically disabling, it does not necessarily cause a high level of disability because people develop coping mechanisms for it. But it is worth recognizing that about two thirds of headache on ≥15 days/month in Russia (as in many other countries) is probably MOH (14), an avoidable condition. Which, with this further and explicit demonstration that headache not only causes much ill health in Russia but costs the country a substantial proportion of its gross domestic product, raises the question of what should be done about all this. The answer is urgent government investment in adequate structured health care for headache, supported by educational initiatives aimed both at health-care professionals and at the general public (who include people affected by headache) (13). The irony, while this does not happen, is that it would almost certainly be cost-saving [24].

The strengths of our study were several. Enquiry into headache yesterday is a powerful approach to population-based burden-of-headache studies, virtually eliminating recall error and any bias that may follow from it. Through face-to-face interviews we achieved a comparatively low non-participation rate of 25.7%, and could avoid the previously described bias of “headache today”: participants with headache on the day they receive the questionnaire by post may defer responding until headache-free, and then spuriously report HY [7]. Since household visits were unannounced and potential respondents were not informed about the survey topic, we believe we had little interest bias (due only to interruption of interviews after they had commenced). Our study sample matched the country population demographically owing to multi-stage cluster sampling. As to limitations, we did not specifically enquire into absenteeism from work yesterday, estimating lost productivity from reports of what had been done in relation to what had been planned. Arguably this served a more conservative analysis; certainly it could not lead to an overestimate of lost productivity at work.

## Conclusion

In conclusion, using HY as a method of enquiry free from recall error, we confirm a very high level of headache-attributed burden in Russia. We repeat our call to the government for action [14], observing that correct action would almost certainly be cost-saving [24].
